# 1,3,5,7,9,11,13,15-Octa­azapenta­cyclo­[9.5.1.1^3,9^.0^6,18^.0^14,17^]octa­decane-4,8,12,16-tetrone monohydrate: a methyl­ene-bridged glycoluril dimer

**DOI:** 10.1107/S160053680802429X

**Published:** 2008-08-20

**Authors:** Pei-Hua Ma, Xin Xiao, Yun-Qian Zhang, Sai-Feng Xue, Zhu Tao

**Affiliations:** a Key Laboratory of Macrocyclic and Supramolecular Chemistry of Guizhou Province, Guizhou University, Guiyang 550025, People’s Republic of China; b Institute of Applied Chemistry, Guizhou University, Guiyang 550025, People’s Republic of China.

## Abstract

In the title compound, C_10_H_12_N_8_O_4_·H_2_O, prepared from the reaction of glycoluril with paraformaldehyde, the organic molecule has *mm* symmetry. The asymmetric unit comprises one quarter of the mol­ecule and a half-mol­ecule of water. The dimer is formed by bridging two glycoluril mol­ecules with methyl­ene groups at the 1 and 6 positions. In the crystal structure, mol­ecules are linked via N—H⋯O and O—H⋯O hydrogen bonds, forming a two-dimensional framework.

## Related literature

For general background, see: Zhao *et al.* (2004 ▶); Zheng *et al.* (2005 ▶).
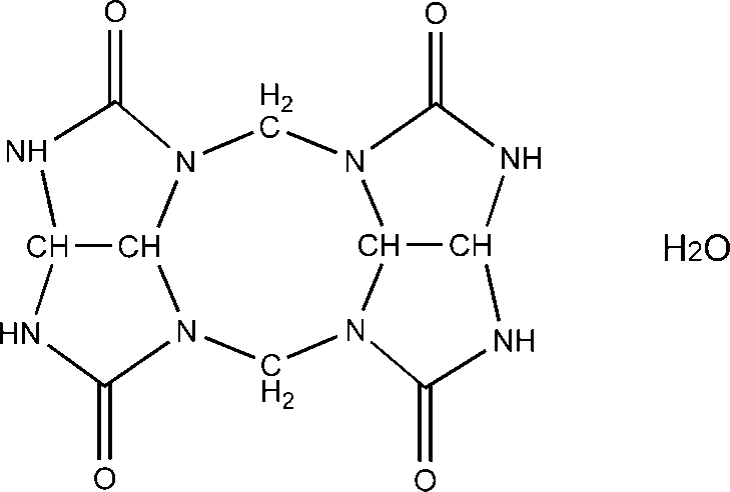


## Experimental

### 

#### Crystal data

C_10_H_12_N_8_O_4_·H_2_O
*M* *_r_* = 326.29
Orthorhombic, 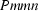

*a* = 10.292 (3) Å
*b* = 12.286 (4) Å
*c* = 4.9530 (15) Å
*V* = 626.2 (3) Å^3^
*Z* = 2
Mo *K*α radiation
μ = 0.14 mm^−1^
*T* = 298 (2) K
0.18 × 0.13 × 0.10 mm

#### Data collection

Bruker APEXII CCD area-detector diffractometer
Absorption correction: multi-scan (*SADABS*; Bruker, 2005 ▶) *T*_min_ = 0.975, *T*_max_ = 0.986
3977 measured reflections
616 independent reflections
528 reflections with *I* > 2σ(*I*)
*R*_int_ = 0.026

#### Refinement

*R*[*F* ^2^ > 2σ(*F* ^2^)] = 0.032
*wR*(*F* ^2^) = 0.089
*S* = 1.11
616 reflections
59 parameters
H-atom parameters constrained
Δρ_max_ = 0.18 e Å^−3^
Δρ_min_ = −0.24 e Å^−3^

### 

Data collection: *APEX2* (Bruker, 2005 ▶); cell refinement: *SAINT* (Bruker, 2005 ▶); data reduction: *SAINT*; program(s) used to solve structure: *SHELXS97* (Sheldrick, 2008 ▶); program(s) used to refine structure: *SHELXL97* (Sheldrick, 2008 ▶); molecular graphics: *ORTEP-3 for Windows* (Farrugia, 1997 ▶); software used to prepare material for publication: *WinGX* (Farrugia, 1999 ▶).

## Supplementary Material

Crystal structure: contains datablocks global, I. DOI: 10.1107/S160053680802429X/sj2521sup1.cif

Structure factors: contains datablocks I. DOI: 10.1107/S160053680802429X/sj2521Isup2.hkl

Additional supplementary materials:  crystallographic information; 3D view; checkCIF report

## Figures and Tables

**Table 1:** Hydrogen-bond geometry (Å, °)

*D*—H⋯*A*	*D*—H	H⋯*A*	*D*⋯*A*	*D*—H⋯*A*
N1—H1⋯O1^i^	0.86	2.01	2.8417 (17)	164
O1*W*—H1*WA*⋯O1^ii^	0.86	2.36	3.0241 (17)	135
O1*W*—H1*WA*⋯O1	0.86	2.36	3.0241 (17)	135

Symmetry codes: (i) 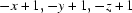; (ii) 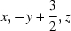.

**Fig. 1.**
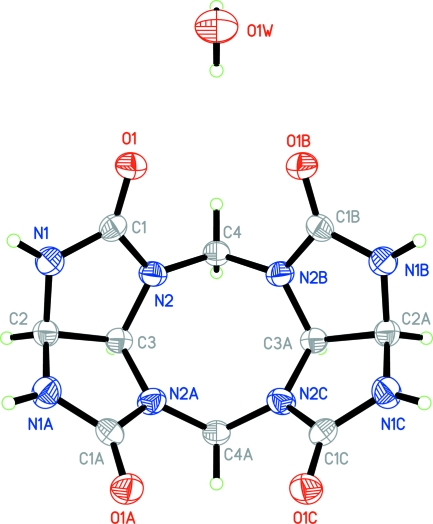
The molecular structure of (I) showing the atom-labelling scheme (Symmetry codes: (A) -x + 3/2, y, z, (B) x, -y + 3/2, z, (C) -x + 3/2, -y + 3/2, z,). Displacement ellipsoids are drawn at the 50% probability level.

**Fig. 2.**
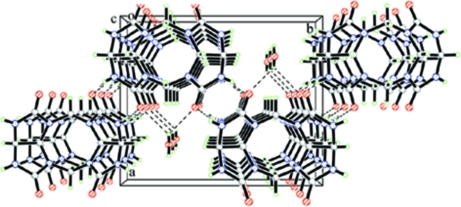
Packing diagram of (I). Hydrogen bonds are shown as dashed lines.

## supplementary crystallographic information

### Comment

In recent years, we have used different alkyl substituted glycolurils and glycoluril dimers as building blocks in the synthesis of partially alkyl substituted cucurbit[*n*]urils In this work, we report the crystal structure of the title compound, a glycoluril dimer, Fig 1.

The molecule comproses two glycoluril units linked by methylene bridges at the 1 and 6 positions. Molecules have mm crystallographic symmetry and the asymmetric unit comprises one quarter of the molecule and a half molecule of water.  In the crystal structure, molecules are linked *via* N1—H1···O1^i^ and O1W—H1WA···O1 hydrogen bonds forming a two-dimensional framework (Table 1 and Fig. 2).

### Experimental

A solution of glycoluril (7.0 g, 0.05 mol) in H_2_SO_4_ (50 ml, 25%) was added to a stirred solution of paraformaldehyde (6.0 g, 0.2 mol) in H_2_SO_4_ (150 ml) and the mixture was kept at 40°C for 5 h. Glycoluril (14.2 g, 0.1 mol) and H_2_SO_4_ (100 ml, 25%) were added in small proportions to this reaction  mixture and the solution held at 80°C on a water bath for 5 h. After cooling  to room temperature, the mixture was filtered to remove the insoluble residue and the filtrate was neutralized with aqueous NH_3_ to pH 7. HCl (150 ml) was then added, the mixture, stirred for 10 min, then filtered again. The solid product was dissolved in 100 ml HCl, and then set aside for three weeks to form colourless crystals of I.

### Refinement

The water H atoms were located in a difference Fourier map and refined as riding on the O atom in these positions with *U*_iso_(H) = 1.2*U*_eq_(O). All other H atoms were placed in calculated positions and refined as riding, with C—H = 0.97 Å (methylene) and 0.98 Å (methine), N—H = 0.86 Å, and *U*_iso_(H) = 1.2*U*_eq_(C,*N*).

### Crystal data

**Table:**

C_10_H_12_N_8_O_4_·H_2_O	*F*_000_ = 340
*M**_r_* = 326.29	*D*_x_ = 1.730 Mg m^−^^3^
Orthorhombic, *P**m**m**n*	Mo *K*α radiation λ = 0.71073 Å
Hall symbol: -P 2ab 2a	Cell parameters from 616 reflections
*a* = 10.292 (3) Å	θ = 2.6–25.1º
*b* = 12.286 (4) Å	µ = 0.14 mm^−^^1^
*c* = 4.9530 (15) Å	*T* = 298 (2) K
*V* = 626.2 (3) Å^3^	Prism, colorless
*Z* = 2	0.18 × 0.13 × 0.10 mm

### Data collection

**Table:**

Bruker APEXII CCD area-detector diffractometer	616 independent reflections
Radiation source: fine-focus sealed tube	528 reflections with *I* > 2σ(*I*)
Monochromator: graphite	*R*_int_ = 0.026
*T* = 298(2) K	θ_max_ = 25.1º
φ and ω scans	θ_min_ = 2.6º
Absorption correction: multi-scan(SADABS; Bruker, 2005)	*h* = −11→12
*T*_min_ = 0.975, *T*_max_ = 0.986	*k* = −14→14
3977 measured reflections	*l* = −5→5

### Refinement

**Table:**

Refinement on *F*^2^	Secondary atom site location: difference Fourier map
Least-squares matrix: full	Hydrogen site location: inferred from neighbouring sites
*R*[*F*^2^ > 2σ(*F*^2^)] = 0.032	H-atom parameters constrained
*wR*(*F*^2^) = 0.089	*w* = 1/[σ^2^(*F*_o_^2^) + (0.049*P*)^2^ + 0.1538*P*] where *P* = (*F*_o_^2^ + 2*F*_c_^2^)/3
*S* = 1.11	(Δ/σ)_max_ < 0.001
616 reflections	Δρ_max_ = 0.18 e Å^−^^3^
59 parameters	Δρ_min_ = −0.24 e Å^−^^3^
Primary atom site location: structure-invariant direct methods	Extinction correction: none

### Special details

**Table:**

Geometry. All e.s.d.'s (except the e.s.d. in the dihedral angle between two l.s. planes) are estimated using the full covariance matrix. The cell e.s.d.'s are taken into account individually in the estimation of e.s.d.'s in distances, angles and torsion angles; correlations between e.s.d.'s in cell parameters are only used when they are defined by crystal symmetry. An approximate (isotropic) treatment of cell e.s.d.'s is used for estimating e.s.d.'s involving l.s. planes.
Refinement. Refinement of *F*^2^ against ALL reflections. The weighted *R*-factor *wR* and goodness of fit *S* are based on *F*^2^, conventional *R*-factors *R* are based on *F*, with *F* set to zero for negative *F*^2^. The threshold expression of *F*^2^ > σ(*F*^2^) is used only for calculating *R*-factors(gt) *etc*. and is not relevant to the choice of reflections for refinement. *R*-factors based on *F*^2^ are statistically about twice as large as those based on *F*, and *R*- factors based on ALL data will be even larger.

### Fractional atomic coordinates and isotropic or equivalent isotropic displacement parameters (Å^2^)

**Table:**

	*x*	*y*	*z*	*U*_iso_*/*U*_eq_	
O1W	0.2500	0.7500	0.5795 (5)	0.0492 (7)	
H1WA	0.3096	0.7500	0.4574	0.059*	
C1	0.56573 (14)	0.59339 (12)	0.2017 (3)	0.0263 (4)	
C2	0.7500	0.49067 (16)	0.0938 (4)	0.0272 (5)	
H2	0.7500	0.4252	−0.0191	0.033*	
C3	0.7500	0.59750 (16)	−0.0774 (4)	0.0254 (5)	
H3	0.7500	0.5819	−0.2714	0.030*	
C4	0.5795 (2)	0.7500	−0.1105 (4)	0.0265 (5)	
H4A	0.5977	0.7500	−0.3027	0.032*	
H4B	0.4859	0.7500	−0.0883	0.032*	
N1	0.63278 (12)	0.50102 (10)	0.2503 (3)	0.0336 (4)	
H1	0.6077	0.4527	0.3646	0.040*	
N2	0.63117 (12)	0.65109 (9)	0.0058 (2)	0.0290 (4)	
O1	0.46406 (10)	0.62196 (9)	0.3073 (2)	0.0341 (3)	

### Atomic displacement parameters (Å^2^)

**Table:**

	*U*^11^	*U*^22^	*U*^33^	*U*^12^	*U*^13^	*U*^23^
O1W	0.0359 (14)	0.0614 (16)	0.0504 (16)	0.000	0.000	0.000
C1	0.0257 (8)	0.0270 (8)	0.0263 (9)	−0.0046 (6)	−0.0020 (6)	−0.0024 (6)
C2	0.0257 (11)	0.0248 (10)	0.0312 (12)	0.000	0.000	−0.0036 (9)
C3	0.0239 (11)	0.0281 (10)	0.0241 (11)	0.000	0.000	−0.0033 (9)
C4	0.0243 (11)	0.0290 (10)	0.0262 (11)	0.000	−0.0049 (8)	0.000
N1	0.0328 (8)	0.0307 (7)	0.0374 (8)	0.0027 (6)	0.0088 (6)	0.0078 (5)
N2	0.0245 (7)	0.0306 (7)	0.0319 (7)	0.0024 (5)	0.0038 (6)	0.0038 (5)
O1	0.0291 (6)	0.0352 (6)	0.0380 (7)	0.0024 (5)	0.0081 (5)	0.0019 (5)

### Geometric parameters (Å, °)

**Table:**

O1W—H1WA	0.8616	C3—N2^i^	1.4487 (16)
C1—O1	1.2214 (17)	C3—N2	1.4487 (16)
C1—N1	1.350 (2)	C3—H3	0.9800
C1—N2	1.3774 (19)	C4—N2^ii^	1.4461 (16)
C2—N1^i^	1.4395 (17)	C4—N2	1.4461 (16)
C2—N1	1.4395 (17)	C4—H4A	0.9700
C2—C3	1.563 (3)	C4—H4B	0.9700
C2—H2	0.9800	N1—H1	0.8600
			
O1—C1—N1	127.08 (14)	C2—C3—H3	111.6
O1—C1—N2	124.95 (14)	N2^ii^—C4—N2	114.35 (17)
N1—C1—N2	107.97 (13)	N2^ii^—C4—H4A	108.7
N1^i^—C2—N1	113.86 (18)	N2—C4—H4A	108.7
N1^i^—C2—C3	102.59 (11)	N2^ii^—C4—H4B	108.7
N1—C2—C3	102.59 (11)	N2—C4—H4B	108.7
N1^i^—C2—H2	112.3	H4A—C4—H4B	107.6
N1—C2—H2	112.3	C1—N1—C2	113.98 (14)
C3—C2—H2	112.3	C1—N1—H1	123.0
N2^i^—C3—N2	115.16 (17)	C2—N1—H1	123.0
N2^i^—C3—C2	103.14 (11)	C1—N2—C4	122.23 (14)
N2—C3—C2	103.14 (11)	C1—N2—C3	112.29 (13)
N2^i^—C3—H3	111.6	C4—N2—C3	125.37 (15)
N2—C3—H3	111.6		
			
N1^i^—C2—C3—N2^i^	0.93 (16)	N1—C1—N2—C4	174.54 (13)
N1—C2—C3—N2^i^	119.26 (13)	O1—C1—N2—C3	179.04 (14)
N1^i^—C2—C3—N2	−119.26 (13)	N1—C1—N2—C3	−1.78 (18)
N1—C2—C3—N2	−0.93 (16)	N2^ii^—C4—N2—C1	98.33 (19)
O1—C1—N1—C2	−179.74 (14)	N2^ii^—C4—N2—C3	−85.8 (2)
N2—C1—N1—C2	1.10 (18)	N2^i^—C3—N2—C1	−109.90 (16)
N1^i^—C2—N1—C1	110.00 (16)	C2—C3—N2—C1	1.67 (17)
C3—C2—N1—C1	−0.06 (18)	N2^i^—C3—N2—C4	73.9 (2)
O1—C1—N2—C4	−4.6 (2)	C2—C3—N2—C4	−174.51 (14)

Symmetry codes: (i) −*x*+3/2, *y*, *z*; (ii) *x*, −*y*+3/2, *z*.

### Hydrogen-bond geometry (Å, °)

**Table:**

*D*—H···*A*	*D*—H	H···*A*	*D*···*A*	*D*—H···*A*
N1—H1···O1^iii^	0.86	2.01	2.8417 (17)	164
O1W—H1WA···O1^ii^	0.86	2.36	3.0241 (17)	135
O1W—H1WA···O1	0.86	2.36	3.0241 (17)	135

Symmetry codes: (iii) −*x*+1, −*y*+1, −*z*+1; (ii) *x*, −*y*+3/2, *z*.
